# An Evaluation of Selected Populations for HIV-1 Vaccine Cohort Development in Nigeria

**DOI:** 10.1371/journal.pone.0166711

**Published:** 2016-12-09

**Authors:** Ogbonnaya S. Njoku, Mark M. Manak, Robert J. O’Connell, Ashley L. W. Shutt, Jennifer A. Malia, Richard A. Heipertz, Sodsai Tovanabutra, Mark J. Milazzo, Gideon Akindiran Akintunde, Abraham S. Alabi, Aminu Suleiman, Amos A. Ogundeji, Terfa S. Kene, Robbie Nelson, Ojor R. Ayemoba, Darrell E. Singer, Merlin L. Robb, Sheila A. Peel, Nelson L. Michael

**Affiliations:** 1 Walter Reed Program, Nigeria, Abuja, Nigeria; 2 Henry Jackson Foundation, MHRP-HJF, Silver Spring, MD, United States of America; 3 Armed Forces Research Institute of Medical Sciences (AFRIMS), Bangkok, Thailand; 4 U.S. Military HIV Research Program, Walter Reed Army Institute of Research, MHRP-WRAIR, Silver Spring, MD, United States of America; 5 CERMEL Albert Schweitzer International Foundation Hospital, Lambarene, Gabon; 6 Nigerian Ministry of Defense Health Implementation Programme, Abuja, Nigeria; 7 Uniformed Services University of Health Sciences (USUHS), Bethesda, MD, United States of America; Cincinnati Children's Hospital Medical Center, UNITED STATES

## Abstract

Development of a globally effective HIV-1 vaccine will need to encompass Nigeria, one of the hardest hit areas, with an estimated 3.2 million people living with HIV. This cross-sectional Institutional Review Board (IRB) approved study was conducted in 2009–12 at four market sites and two highway settlements sites in Nigeria to identify and characterize populations at high risk for HIV; engage support of local stakeholders; and assess the level of interest in future vaccine studies. Demographic, HIV risk data were collected by structured interviewer-administered questionnaires. Blood samples were tested on site by HIV rapid diagnostic tests, followed by rigorous confirmatory testing, subtype evaluation and testing for HBV and HCV markers in a clinical reference laboratory. Of 3229 study participants, 326 were HIV infected as confirmed by Western Blot or RNA, with a HIV prevalence of 15.4%-23.9% at highway settlements and 3.1%-9.1% at market sites. There was no observable correlation of prevalence of HIV-1 (10.1%) with HBV (10.9%) or HCV (2.9%). Major HIV-1 subtypes included CRF02_AG (37.5%); G (27.5%); G/CRF02_AG (25.9%); and non-typeable (8.9%), with 0.3% HIV-2. Univariate analysis found age, gender, marital status, level of education, and sex under substance influence as significant risk factors for HIV (p<0.001). Educating and winning the trust of local community leadership ensured high level of participation (53.3–77.9%) and willingness to participate in future studies (95%). The high HIV prevalence and high risk of HIV infection at highway settlement and mammy markets make them well suited for targeting future vaccine trials in Nigeria.

## Introduction

A recent UNAIDS report for 2014 estimated that 36.9 million people worldwide were living with HIV including 2.0 million people newly infected in 2014 [1]. Hardest hit by the HIV/AIDS pandemic is sub-Saharan Africa where the disease continues to devastate communities, thereby rolling back decades of economic development [2, 3]. Nigeria, the most populous country in this region has an estimated 3.2 million people living with HIV disease, (national HIV prevalence of 3.4%), and carries the second highest burden of HIV/AIDS in the world [1]. Despite the downward trend in overall HIV prevalence, moderate to severe epidemics persist in the 36 States and Federal Capital Territory of Nigeria, with over 10% prevalence in the general population in some States [2, 4].

Durable elimination of the HIV pandemic will require new combination prevention strategies including development of a globally effective vaccine, coupled with other proven biomedical and behavioral interventions [5]. Previous and on-going vaccine studies by the US Military HIV Research Program (MHRP) had been in Thailand where the epidemic is mainly due to HIV-1 subtype CRF01_AE and in East Africa dominated by subtypes A, C, D and intersubtype recombinants. These strategies will need to be expanded to protect against other subtypes and account for other regions such as West Africa with its unique HIV-1 circulating subtypes [6–10].

Some research studies previously conducted in Nigeria, such as the failed Pfizer trials of 1996, lacked the required IRB regulatory approvals and documented evidence of informed consent [11–13]. Any future trials and vaccine studies, therefore, must ensure appropriate community engagement, site preparedness and adherence to the principles of Good Participatory Practice (GPP) to restore the trust of the community [14]. The aim of this study was to identify populations with high HIV prevalence and at high risk for HIV infection for vaccine cohort development in Nigeria; assess the level of interest of populations to participate; and, engage support of local stakeholders. Specific objectives of the study included estimation of the distribution of HIV subtypes and prevalence of Hepatitis B and C in the study population. Follow up incidence studies in key populations at risk for HIV infection will further inform vaccine development in Nigeria.

## Methods

This cross-sectional observational study commenced in 2009 and was initiated at six sites (Makurdi, Abuja, Enugu, Kaduna, Tafa and Ojo-Lagos) distributed across four States and the Federal Capital Territory of Nigeria. The initial population evaluated focused on market sites in proximity of selected military hospitals and PEPFAR Centers including “mammy markets” run by civilians, and neighboring bars, hotels, and restaurants, where many people congregate with opportunities for social interactions. Two additional sites at highway settlements were later added to provide access to populations at higher risk of HIV infection. Gaining approval from civil authorities at the Federal, State and Local levels, and from community and military leaders prior to study implementation at each site helped population acceptance of the study. While the institutional partners provided general awareness, legitimacy, and letters of support, community leaders and community associations were instrumental in the mobilization of study participants.

The field study commenced with advocacy visits, key informant interviews, and focus group discussions. The owners and managers of businesses in all the fixed structures to include shops, bars, and guesthouses in the selected study area were asked to fill out a questionnaire about employees working at their premises. The socio-demographic information of approximately 15,400 individuals identified from this initial questionnaire was entered into a database, and a computerized random number generator was used to select approximately 25% of the male and female workers between 18 and 40 years of age (550 volunteers per site) for participation in the cross-sectional study. The randomly selected volunteers, which included food sellers, hotel workers, bar workers, and other support staff were visited at their workplace during the afternoon prior to the planned day of participation. They were informed of the study and invited to come to the study site the following morning. After obtaining a signed informed consent, a structured interviewer administered questionnaire was administered to the enrolled volunteers to collect demographic, behavioral, and HIV knowledge data that were captured with palm-held devices, then transfer into the study database. The volunteers in the cross-sectional study were then offered HIV counseling, testing, and a voluntary blood draw.

After study initiation, the original protocol was amended to include additional populations at increased risk of HIV infection. Two highway settlement sites were added to represent higher risk populations including commercial sex workers (CSWs) aged 18 to 50 [15]. The sexual activity questions at these sites narrowed to focus on the most recent 3 months and additional risk factor questions were added. Blood samples were collected for immediate field HIV rapid diagnostic testing using Determine and Stat-Pak Rapid Test Kits in a parallel algorithm, with UniGold Rapid test kits as tie-breaker for discordant results, according to existing National Guidelines [16, 17]. Separate specimens were collected from each study participant for additional comprehensive reference laboratory testing at the HIV Diagnostics and Reference Laboratory, U.S. Military HIV Research Program (MHRP), Walter Reed Army Institute of Research (WRAIR) (Silver Spring, MD, USA). Samples reactive by GS HIV-1/2/O (HIV EIA) (Bio-Rad Laboratories, Inc Redmond, WA) were repeated in duplicate; repeat reactive (RR) samples then reflexed to GS HIV-1 Western Blot (WB) and MultiSpot (MS) HIV-1/HIV-2 Rapid Test assays (Bio-Rad Laboratories, Inc, Redmond, WA). WB Positive (POS) or Indeterminate (IND) samples were subjected to HIV-1 RNA analysis by either APTIMA HIV-1 RNA Qualitative Assay (Hologic, San Diego, CA) or the quantitative Abbott RealTime HIV-1 assay (Abbott, Chicago, IL).

The prevalence of HIV infection at each site was determined by the percentage of participants who were EIA RR with confirmation by the HIV-1 Western Blot or HIV-1 RNA. Samples with VL of ≥1,000 copies/ml were referred to the MHRP Molecular Sequencing Core Laboratory for analysis by multi-region hybridization assays (MHA) specially developed to identify HIV-1 subtypes CRF02_AG, G and recombinants [18]. A single sample demonstrating HIV-2 reactivity on the MultiSpot assay was further analyzed by a HIV-2 Quantitative RNA Laboratory Developed Assay with a detection sensitivity of 100 copies/ml.

All study samples were screened for Hepatitis B Virus infection by antibody to HBV surface antigen (Monolisa Anti-HBs EIA), antibody to core antigen (Monolisa Anti-HBc EIA), and Hepatitis B surface antigen (Genscreen HBsAg 3.0 EIA) assays (Bio-Rad Laboratories, Redmond, WA). HCV testing was performed with the Ortho Chiron HCV Version 3.0 ELISA Test System (Ortho-Clinical Diagnostics, Inc, Rartin, NJ), with RR samples reflexed to the Chiron HCV RIBA 3.0 Strip Immunoblot Assay (SIA) (Novartis Vaccines and Diagnostics), or to the Inno-LIA HCV Score (Fujirebio Europe, Belgium).

Statistical methods used in analysis of risk factors included univariate analyses using Chi-square test (or Fisher’s exact test when cell frequencies were < = 5) for categorical variables and the Student-t test for continuous variables with normal distribution. Unadjusted odds ratios were calculated for selected risk behavioral factors. Data analysis was performed using SAS version 9.4 (SAS Institute, Cary, NC USA).

## Ethical Considerations

This study (WRAIR#1476, RV 230) was approved by the Institutional Review Board (IRB) of the Walter Reed Army Institute of Research (WRAIR) and the National Health Research Ethics Committee of Nigeria (NHREC). All participants underwent an Informed Consent procedure, signed, and received a copy of their respective Informed Consent Form (ICF). The original signed ICF was retained within regulatory files. HIV counseling and testing was offered after baseline interview. Specimen transfer was per NHREC approved materials transfer agreement (MTA). President's Emergency Plan for AIDS Relief (PEPFAR) health facilities offering HIV prevention and anti-retroviral treatment services close to the study sites were available for comprehensive care of all eligible study participants at no cost.

## Results

**Fig 1:**
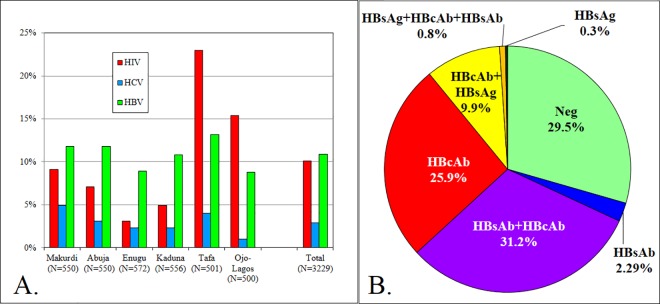
Distribution of HIV, HCV and HBV infections among 3229 volunteers tested. A. Percent HIV, HCV and HBV infections at each site. B. Distribution of HBV markers in population studied. 68.8% of the population was positive for at least one marker of HBV infection.

Of the 3229 volunteers who completed the study at the six study sites, 326 were HIV infected as confirmed by Western Blot or RNA detection (16). The mean overall HIV-1 point prevalence across the six sites was 10.1% (95% CI 9.1%-11.1%). The highest prevalence was detected at the two highway settlements sites, Tafa 23.0% (19.3%-26.6%) and Ojo-Lagos 15.4% (12.2%-18.6%), while the market sites ranged from 3.1% (1.7%-4.6%) in Enugu to 9.1% (6.7%-11.5%) in Makurdi. Of the 326 samples confirmed as HIV positive, only one sample (from Abuja) was HIV-2 positive, representing 0.3% of all HIV infections in our study population. The percentages of confirmed HIV, HCV and HBV infections at each site are shown in Fig 1A, with the distribution of HBV markers shown in Fig 1B. HBsAg, a marker of current or ongoing infection was used as a measure of HBV prevalence, at 10.9% (9.9%-12.0%). The mean point prevalence for HCV was 2.9% (2.4%-3.6%) ranging from 1.0% (0.1%-1.9%) in Ojo-Lagos site to 4.9% (3.1%-6.7%) in Makurdi. HBV point prevalence was higher than that of HCV in all sites with the highest, 13.2%, in Tafa (10.2–16.1%) and the lowest, 8.8% in Ojo-Lagos (6.6%-11.3%). No correlations among the prevalence rates of the three diseases (HIV, HCV and HBV), as assessed by Pearson product-moment and Spearman rank-order correlations, were found (data not shown).

HIV genotyping by MHA was performed on HIV-1 positive samples having a viral load ≥ 1,000 copies/mL (230 of 326, 70.6%). The distribution of the major HIV-1 subtypes across all six sites were 37.5% CRF02_AG; 27.7% G; 25.9% G/CRF02_AG; and 8.9% non-typeable (Table 1). An in-depth molecular phylogenetic analysis of sequences from volunteers in this study is reported elsewhere [57].

**Table 1:** Distribution of major HIV-1 subtypes in the six study sites as analyzed by Multi-region Hybridization Assay (MHA).

	CRF02_AG		G		G/CRF02_AG		Other		Total	
**Makurdi**	12	33.3%	7	19.4%	7	19.4%	4	11.1%	30	100%
**Abuja**	7	38.9%	5	27.8%	4	22.2%	2	11.1%	18	100%
**Enugu**	3	27.3%	4	36.4%	2	18.2%	2	18.2%	11	100%
**Kaduna**	8	66.7%	2	16.7%	1	8.3%	1	8.3%	12	100%
**Tafa**	27	28.4%	34	35.8%	29	30.5%	5	5.3%	95	100%
**Ojo-Lagos**	27	46.6%	10	17.2%	15	25.9%	6	10.3%	58	100%
**Total**	**84**	**37.5%**	**62**	**27.7%**	**58**	**25.9%**	**20**	**8.9%**	**224**	**100%**

Univariate analyses of potential risk factors for HIV infection in the market sites (Makurdi, Abuja, Enugu and Kaduna) and the highway settlements (Tafa and Ojo-Lagos) are shown in Tables 2 and 3. Age, gender and level of education were found to be significant risk factors in both populations (p<0.001). Females between 18–30 years were more than three times more likely to be HIV infected than males. The highest prevalence in males (14.2%) was in those over 30 years of age. Males constituted 62.7% of volunteers below the age of 21 in the market sites, but none was identified as HIV infected. Volunteers with only primary school education had a significantly higher HIV risk compared to those who completed secondary school or higher. The number of sexual partners in the past year did not show a significant correlation with HIV infection in the market population, with the majority of infected participants claiming to have only a single partner (p = 0.088). The overall level of reported transactional sex was low, but those who admitted to receipt of goods for sex were 2.8 times more likely (5.2% vs. 1.8%) to be HIV infected than those who did not (p<0.001). Bartering for sex did not show increased HIV risk (3.7% vs. 6.1%, odds ratio 0.565 with 95% CI 0.239–1.481). History of previous HIV testing correlated significantly with HIV status (p = 0.008).

**Table 2:** Univariate Analysis of HIV Risk Factors at the various study sites. The Highway Settlement sites were added after study initiation, and included more risk factors and focused sexual activity questions only on the past 3 months.

		Market Sites			Highway Settlements		
Risk Factor	Status	HIV Positive	HIV Negative	p Value	HIV Positive	HIV Negative	p Value
		134 (6.0%)	2094 (94.0%)		192 (19.2%)	809 (80.80%)	
**Age (years)**	mean (SD)	29.8 (5.2)	26.5 (6.1)	< 0.001	28.6 (6.0)	27.5 (7.1)	0.034
	range	19–40	18–40		18–45	18–50	
**Age Group**	Under 21	3 (2.2%)	396 (18.9%)	< 0.001	10 (5.2%)	109 (13.4%)	0.0015
	21–30	79 (59.0%)	1194 (56.8%)		128 (66.7%)	512 (62.8%)	
	31–40	52 (38.8%)	511 (24.3%)		47 (24.5%)	142 (17.4%)	
	> 40	0 (0%)	0 (0%)		7 (3.7%)	53 (6.5%)	
**Age/Gender**	Female < 21	3 (2.2%)	146 (7.0%)	< 0.001	10 (5.2%)	83 (10.3%)	< 0.001
	Female 21–30	68 (50.8%)	494 (23.6%)		120 (62.5%)	306 (37.8%)	
	Female > 30	33 (24.6%)	206 (9.8%)		49 (25.5%)	120(14.8%)	
	Male < 21	0 (0%)	250 (11.9%)		0 (0%)	25 (3.1%)	
	Male 21–30	11 (8.2%)	696 (33.2%)		8 (4.2%)	203 (25.1%)	
	Male > 30	19 (14.2%)	302 (14.5%)		5 (2.6%)	72 (8.9%)	
**Gender**	Male	30 (22.4%)	1251 (59.5%)	<0.001	13 (6.8%)	301 (36.9%)	<0.001
	Female	104 (77.6%)	850 (40.5%)		179 (93.2%)	515 (63.1%)	
**Education**	Incomplete primary	14 (10.5%)	104 (5.0%)	<0.001	42 (21.9%)	123 (15.3%)	<0.001
	Completed Primary	52 (38.8%)	612 (29.2%)		106 (55.2%)	329 (40.7%)	
	Completed Secondary	68 (50.7%)	1378 (65.8%)		44 (22.9%)	357 (44.1%)	
**Ever Tested for HIV**	No	66 (49.3%)	1314 (62.8%)	0.008	74 (38.5%)	386 (47.7%)	0.044
	Yes	68 (50.8%)	780 (37.2%)		118 (61.5%)	423 (52.3%)	
** **		In the Past Year			In the Past 3 Months		
**Number of Sexual Partners**	Mean (SD)	3.47 (11.2)	1.80 (2.8)	0.088	83.5 (128.7)	79.4 (150.6)	0.721
	Range	0–99	0–52		0–504	0–900	
	None	11 (8.2%)	414 (19.7%)		13 (6.8%)	110 (13.6%)	
	1 partner	79 (59.0%)	1061 (50.7%)		27 (14.1%)	220 (27.2%)	
	>1 partner	44 (32.8%)	619 (29.6%)		152 (79.2%)	479 (59.2%)	
**Received Anything for Sex**	No	127 (94.8%)	2058 (98.3%)	<0.001	45 (23.4%)	426 (52.7%)	<0.001
	Yes	7 (5.2%)	36 (1.7%)		147 (76.6%)	383 (47.3%)	
**Gave Anything for Sex**	No	129 (96.3%)	1966 (93.9%)	<0.001	179 (93.2%)	681 (84.2%)	<0.001
	Yes	5 (3.7%)	128 (6.1%)		13 (6.8%)	128 (15.8%)	
**Occupation**	Sex work				127 (66.2%)	300 (37.1%)	< 0.001
	Bar work				19 (9.9%)	185 (22.9%)	
	Food seller				35 (18.2%)	177 (21.9%)	
	Other				11 (5.7%)	147 (18.2%)	
**Ever had sex with someone of same sex**	No				189 (98.4%)	765 (94.6%)	0.058
	Yes				2 (1.0%)	17 (2.1%)	
	Refused/Don’t Know				1 (0.5%)	27 (3.3%)	
**Ever had sex under influence of drugs**	No				106 (55.2%)	536 (66.3%)	< 0.001
	Yes		Not		86 (44.8%)	248 (30.7%)	
	Refused/Don’t Know		Asked		0 (0%)	25 (3.1%)	
**Injection Drug Use**	No				186 (96.9%)	794 (98.2%)	0.261
	Yes				6 (3.1%)	15 (1.9%)	
	Refused/Don’t Know				0 (0%)	0 (0%)	
**Marital Status**	Single				67 (34.9%)	442 (54.6%)	<0.001
	Married				16 (8.3%)	172 (21.3%)	
	Divorced				43 (22.4%)	67 (8.3%)	
	Separated				59 (30.7%)	103 (12.7%)	
	Living Together				7 (3.7%)	25 (3.1%)	

**Table 3:** Unadjusted Odds Ratios for Selected Behavioral Risk Factors.

	Market Sites			Highway Settlements		
Risk Factor	Odds Ratio	95% CI	p Value	Odds Ratio	95% CI	p Value
**Number of Sexual Partners**	1.065	0.802–1.414	0.664	1.79	1.347–2.378	< 0.001
**Received Anything for Sex**	3.151	1.375–7.220	0.007	3.633	2.531–5.214	< 0.001
**Gave Anything for Sex**	0.595	0.239–1.481	0.265	0.386	0.213–0.700	0.002
**Ever had sex with someone of same sex**	Not Asked			2.079	0.597–7.239	0.250
**Ever had sex under influence of drugs**				0.570	0.413–0.787	< 0.001
**Injection Drug Use**				0.586	0.224–1.530	0.275

A similar analysis of HIV risk factors in the highway settlement sites also indicated age, gender, and educational attainment as statistically significant risk factors. The vast majority (93.2%) of all HIV infected participants in the highway settlements were females with highest HIV prevalence within those 21 to 30 years of age. No males below the age of 21 were found to be seropositive for HIV. Those who did not complete primary school demonstrated a significantly higher HIV risk compared to those who did (p<0.001). Divorced (22.4%) and separated (30.7%) individuals were more likely to be HIV infected than those who were married (8.3%) or living together (3.7%). Commercial Sex Work (66.2%) as a means of livelihood was associated with higher HIV risk compared to food selling (18.2%) and bar work (9.9%). About one third (33.4%) of all study participants reported sexual encounters under the influence of drugs. Alcohol was the most common substance consumed (73.7%) followed by cannabis (41.0%). Those who reported sexual intercourse under substance influence had a statistically significantly higher risk for HIV infection compared to those who did not (p < 0.001). Of HIV infected individuals, 79.2% admitted to having more than one partner. Many (76.6%) HIV infected participants admitted to receiving goods for sex—a significant risk factor (p < 0.001). In contrast, providing goods for sex (6.8% of infected participants versus 15.8% of uninfected participants) did not increase infection risk. History of previous HIV testing correlated positively with HIV status in both market and highway settlement sites (p = 0.044).

**Fig 2:**
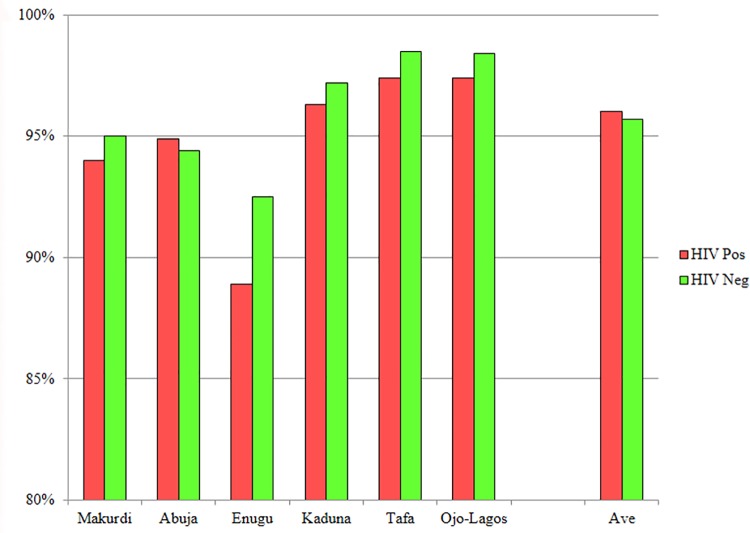
Willingness of volunteers to participate in future studies.

The percentage of eligible persons who agreed to participate in this cross-sectional study ranged from 53.3% in Abuja to 77.9% in Kaduna. A high percentage of HIV infected (96.0%) and HIV non-infected (95.7%) volunteers expressed willingness to participate (WTP) in future studies involving regular blood draws (Fig 2). Over 50% of participants, however, indicated “unknown” concerning planned length of stay at their present location: 36% at Ojo-Lagos, 55% in Makurdi and 72% in Tafa. Only 49% of participants at the Ojo-Lagos site, 23% at Tafa, and 21% at Kaduna indicated they intended to remain within their locale for one year or less; while 24% of participants at the Makurdi, 20% at Enugu, and 4% at Tafa planned to stay more than 2 years in their location. The mobility of populations studied will need to be considered when planning clinical trials as transient populations impact retention and success of studies requiring long term commitments from participants.

## Discussion

This cross-sectional, observational study sought to identify populations and locations with favorable epidemiological and logistic characteristics for cohort development for HIV vaccine studies in Nigeria. Site selection and sample size for HIV-1 preventative efficacy trials in high-risk volunteers depends heavily on HIV-1 incidence, retention, and willingness to participate in future trials. This study provides useful data to characterize six potential geographically diverse trial sites. The first four sites were mammy/general markets contiguous to Nigerian military installations and PEPFAR treatment facilities in Makurdi, Abuja, Enugu, and Kaduna where the observed HIV point prevalence closely mirrored that of the general population [19]. Although HIV incidence at these sites was not known, the point prevalence (3.1–9.1%) indicates a threshold of 3% incidence per year considered optimal for HIV vaccine efficacy trials is unlikely. We therefore sought to include a higher risk population, commercial sex workers (CSWs) known to interact socially with long distance truck drivers in highway settlement communities; thus, expected to be at increased risk of HIV infection [20–23]. The two highway settlements in our study, Tafa and Ojo-Lagos, demonstrated the highest overall HIV prevalence of 23.0% and 15.4% respectively, and are therefore more suitable than the market sites for HIV incidence and vaccine studies.

Of the 326 HIV infections confirmed in 3,229 participants in this study, only a single HIV-2 (0.3% of all HIV infections) was identified, consistent with more recent studies showing a decline of HIV-2 prevalence in West Africa [24]. While a serological survey conducted in the mid-1990s indicated that HIV-2 was responsible for 4.5% of all HIV infections in Nigeria [25], lack of PCR confirmation likely resulted in overestimation of HIV-2 prevalence [26]. More recent studies of long distance truck drivers (N = 100) in the Niger Delta Zone of Nigeria indicated a 10% HIV prevalence in this population, with only one HIV-2 infection (1%) identified, but not confirmed [23]. Forbi et al [27] reported 18 HIV-2 infections (5.4%) out of 335 HIV infected female sex workers. Of the 18 HIV-2 cases, 17 were co-infected with HIV-1.

Molecular analysis of 224 HIV positive samples indicated that CRF02_AG, G/CRF02_AG and G were major circulating recombinant forms and subtypes, respectively in our study [27]. This analysis may represent one of the largest HIV genotypic analyses at the population level across Nigeria. These are in agreement with other HIV-1 subtype studies in this region [6–10, 28–30].

Our findings of disproportionate numbers of HIV infections among young women in Nigeria is consistent with previous reports from sub-Saharan Africa where young women are three to four times more likely to be HIV-infected than their male peers [2, 31–34]. Factors contributing to the higher HIV burden in females may include the two to three fold increased likelihood of heterosexual transmission of HIV from a man to a woman, and even higher transmission risk during forced or coerced sex, which is common but rarely reported by women [35, 36]. The finding of low level of education as a risk factor in HIV spread is corroborated by a survey of sexual practices among long distance truck drivers in Nigeria, which found that drivers with less than secondary education were less likely to use condoms [37]. Whereas the female brothel-based sex workers in the two highway sites demonstrated the highest seropositivity (29.6%), it is interesting to note that food sellers and bar workers at the highway sites, 16.0% and 9.3%, respectively, were of higher HIV prevalence than market workers in Makurdi (9.1%), the market site with the highest prevalence. Additional risk associations in highway populations included marital status and substance abuse. About one-third (33.4%) of all study participants in the highway sites reported sex under the influence of drugs, with alcohol (73.3%) and cannabis (40.8%) being the most common substances used, consistent with other studies in African cities [37–39]. Correlation of risk factors with prevalence may guide selection of most at risk sub-populations based on demographic and risk behavior questionnaire results for enrollment into the study.

The high endemic HBV (10.9%) and low HCV (2.9%) prevalence observed in our study are consistent with previous studies of HBV and HCV in Nigeria which were based on screening of hospital patients, blood donors or HIV-infected populations [40–45]. A survey of pregnant women attending hospital clinics in Benin City, Nigeria, found HBV prevalence at 12.5% and HCV at 3.6%. One of our two highway sites, Tafa, with the highest HIV prevalence also has the highest HBV prevalence (13.2%) while the other, (Ojo-Lagos) has the lowest prevalence of HCV (1.0%). Overall, there are no clear correlations between these three viral infections in the sites studied. The high percent of anti-HBc reactive (65.6%) and anti-HBs reactive (32.1%) participants is evidence of prior infection, and indicative that HBV infection is truly endemic in Nigeria (Fig 2). The high percentage of participants with detectable HBV antibodies in this study will need to be considered in selecting volunteers for future vaccine studies.

Earlier HIV Vaccine preparedness studies (VPS) demonstrated that community involvement is critical in attaining confidence of populations at large. Encouragement from family and friends was shown to be important in successful recruitment and retention of study volunteers [46, 47]. Important motivators for participation included an altruistic desire to contribute to protection from HIV, prevention, eliminating the spread of HIV/AIDS, and improved access to health care services and counseling [48, 49]. Barriers to participation included safety concerns, mistrust or misunderstanding of study design, and fear of social stigmatization [50]. In the present study, educational programs as well as programs focused upon attaining trust of the market association leadership were key to successful recruitment of volunteers at market sites. In the two highway settlements, our community-based efforts included engagement of existing youth development or health-focused community organizations that had been previously active in health awareness activities. Similar levels of engagement were reported by Kiwanuka and colleagues among fishing communities in Uganda [45]. Overall, 68.2% of eligible persons in the census populations in the six study sites accepted our invitation to enroll and participate in the cross-sectional study. We have shown that conducting successful population based research studies in Nigeria requires a high level of advocacy, community engagement, and co-operation among all stakeholders. Furthermore, committed community leadership is imperative for effective sensitization of the targeted populations. The high levels (97–98%) of willingness of volunteers to participate in future studies at the last three sites (Kaduna, Tafa and Ojo-Lagos) may have been due to improved community mobilization skills of our study team. The rates of willingness to participate in future vaccine trials recorded in this study compares favorably with similar studies in Sub Saharan Africa [51–53].

The high rate of mobility among the populations in our study, however, may present a major challenge to retention in a future cohort study. Study population instability due to mobility may be minimized by adequate compensation for travel expenses to encourage longer retention and improvement in follow-up rates of high risk cohorts [54, 55, 56]. Careful consideration of fair, but non coercive financial or other incentives, however, should be balanced within cultural and ethical contexts. Based on successful community mobilization in this study, it is expected that with reasonable compensations for travel and communication costs in this era of almost universal access to cell phones, retention of cohorts even among highly mobile populations will be enhanced.

A limitation of our study is that following a mid-study review, it was determined that the initial target population in the market areas demonstrated a lower HIV prevalence than anticipated. The protocol was then amended to include known high risk populations, CSWs and bar workers, in two highway settlement sites (Tafa and Ojo-Lagos). Changes in enrollment criteria for these two highway settlement sites with a 3 month rather a 12 month time frame for some of the sexual risk questions and addition of more risk factors made data analysis of the behavioral questionnaire more challenging. Specifying brothel-based CSW, hotel, bar workers, and food sellers in the highway settlements may have excluded most of the male sexual clients of younger women in the study, skewed the population toward younger women, and could explain the discrepancies between ‘receiving something for sex’ and ‘giving something for sex’ in terms of HIV risk.

## Conclusions

Our study has demonstrated that highway settlement communities may be suitable for vaccine cohort development in Nigeria. Additionally, young females working in brothels, bars, or involved in food selling in these sites should be targeted for prevention messaging. Age, gender, occupation, and level of education are significant risk factors for HIV infection in these communities. Our study has vividly demonstrated high HIV-1 prevalence in two highway settlements in Nigeria. This finding, along with the high percentage of persons willing to participate in future research studies should accelerate the process of preparation of selected communities for future biomedical prevention interventions in Nigeria, to include HIV-1 vaccine trials among identified high risk populations.
